# Prosthesis extrusion post total ossicular replacement ossiculoplasty (TORP) following isotretinoin use: A case report and literature review of peri-operative isotretinoin safety

**DOI:** 10.1016/j.amsu.2022.104469

**Published:** 2022-08-25

**Authors:** Mohammed Alwabili, Nour Alotaibi, Saleh Alamry

**Affiliations:** a Department Of Otolaryngology Head And Neck Surgery, Prince Sultan Military Medical City, Riyadh, Saudi Arabia; b Collage of Medicine, Dar Al Uloom University, Riyadh, Saudi Arabia

**Keywords:** Prosthesis extrusion, Isotretinoin, Ossicular replacement prosthesis (TORP), Acne, Ossiculoplasty, Case report

## Abstract

Ossiculplasty is the preferred intervention to restore the mechanism of sound transmission in patients with ossicular deformities. Here, we present a case of a young female who underwent cartilage tympanoplasty with total ossicular replacement prosthesis (TORP) to the right ear. Her recovery was progressing well with signs of postoperative improvements for almost two years until she was started on Isotretinoin 40 mg by her dermatologist. A few months later, she presented with worsening hearing loss and bloody discharge to the same ear. Consequently, examination showed that part of the prosthetic device was extruding through the cartilage graft, with signs of graft thinning and documented conductive hearing loss. Although it is evident that isotretinoin has an impact on various healing processes. Literature varies on the recommendations on the use of isotretinoin before and after surgical procedures and the exact magnitude of impact is still to be determined. Our case suggests that using oral Isotretinoin may lead to the thinning of cartilage graft and thus extruding of the prosthesis. Clinicians must be aware of the possible adverse associations of oral Isotretinoin to healing, especially surgeries involving extremely delicate skin or cartilage grafts like in our case.

## Highlights

• Isotretinoin has an impact on various healing processes.
• Literature varies on the recommendations on the use of isotretinoin before and after surgical procedures.
• Isotretinoin may lead to the thinning of cartilage graft and thus extruding of the prosthesis.
• Clinicians must be aware of the possible adverse associations of Isotretinoin to healing, especially surgeries involving extremely delicate skin or cartilage grafts.

## 1 Introduction and importance

Ossiculoplasty is referred to as the surgical intervention in which ossicles (malleus, incus, and stapes) bones are reconstructed to restore the mechanism of sound transmission to the inner ear from the tympanic membrane [1]. It is considered the core intervention for diseases causing ossicular chain abnormalities such as fixation or discontinuity. Ossicuplasty was initially attempted by Hall and Ryztner in 1957 using autografted ossicles and had since evolved with various recent advancements [2]. The surgical technique of ossiculoplasty performed using total ossicular replacement prostheses (TORP) is considered when the patient doesn't have malleus, incus, and stapes supra structures. It is commonly operated using interposed cartilage between the prostheses device and the tympanic membrane [3]. As a result, the use of cartilage graft has been shown to decrease surgery failure and prostheses extrusion [1,4]. Since titanium prostheses were introduced in the 1990s, they have been widely utilized in ossiculoplasty due to their biological compatibility features to the human ear, availability, and lightweight [5]. Regardless of prostheses type or ossiculoplasty technique, failure of such interventions has been reported and continuously investigated and attributed to a variety of contributing factors. Here, we report a case of a young female who underwent ossiculoplasty with TORP and then immediately started on isotretinoin acid for her acne which resulted in what we believe to be cartilage graft thinning and prostheses extrusion. We believe surgeons who commonly perform surgeries with extreamally delicate skin or cartilage grafts should be aware of isotretinoin possible effect. This case report has been reported in line with the SCARE Criteria [6].

## 2 Case presentation

**Fig. 1:**
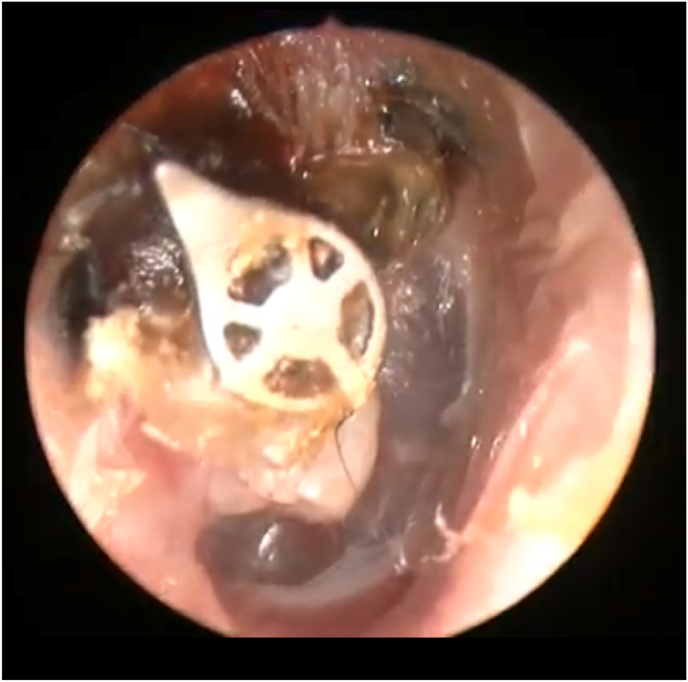
otoscopic view of right tympanic membrane showing extrusion of prosthesis device with cartilage graft thinning.

We present a case of a 25-year-old female with unremarkable past medical or surgical history who regularly following in our clinic at Prince Sultan Military Medical City (PSMMC) in Riyadh, Saudi Arabia as adhesive otitis media. She initially suffered from right ear pain for more than three years. On clinical examination, she presented with chronic tympanic membrane perforations that extended to the ossicles leading to ossicular extrusions with no chronic otorrhea. After thorough investigations and consoling, she agreed to undergo cartilage tympanoplasty with total ossicular replacement prosthesis (TORP) on the 7^th^ of April 2018 to the right ear under general anesthesia. The surgery done in our institution in a well controlled settings with more than 20 years experience in performing similar surgeries. Her postoperative period was progressing well without any significant complaints. Additionally, pure-tone audiometry (PTA) examination showed air-bone gap closure indicating favorable objective post-operative improvements. She was genuinely thrilled with the outcome of her surgery and was closely compliant with our post operative instructions and follow up in our clinic. However, two years following surgery, she started on daily Isotretinoin 40 mg for acne treatment as prescribed by her dermatologist. A few months after initiating her Isotretinoin therapy, she presented as a “walk-in” to our clinic with worsening hearing loss and bloody discharge from her operated ear. When we revised her medical history, she admits that her complaints started after her dermatologist increased the dose of isotretinoin from 20 to 40 mg daily. During her period of worsening symptoms, she didn't report any fever or frequent visits to emergency. Denies any further attacks of ear infection or trauma. Further on, there was no vertigo, tinnitus, or facial weakness. The otoscopy to the right ear showed the prosthesis device was in its place, while part of it was extruding through a cartilage graft. However, the graft was intact, and there was mild bloody to whitish discharge coming from the middle ear [Fig. 1]. Additionally, apparent thinning of cartilage graft with prosthesis tips can be seen through the graft. Rinne test was negative on the right side and weber was lateralizing to right. Patient was medically free with no past surgical or drug history and unremarkable social or family history.

## 3 Discussion

We believe this case represent a relevance to otolaryngologist, otologist and other surgeons who commonly perform surgeries involving delicate skin or cartilage grafts. Additional importance applied to general practitioners and dermatologist who in fact prescribe and follow patient on Isotretinoin therapy. Isotretinoin is an oral medication developed by Hoffman-La Roche (Roche) in 1982 for the treatment of severe, persistent, and nodular acne [7]. Despite the lack of data on the prescription rate for oral isotretinoin, it is believed that its use has been increased considerably in recent years. Further data revealed increased use of isotretinoin for mild to moderate cases of acne [8]. From literature to the best of our knowledge, no previous study reported a possible association between isotretinoin use and cartilage graft thinning or prosthesis extrusion complications following cartilage tympanoplasty and TORP. Isotretinoin's side effects are well-documented, and they affect almost organ in the human body. Many studies reported possible adverse events between isotretinoin administration and postoperative healing involving many organ systems including skin, cartilage, bone, skeletal muscle, and others [9]. In addition, many articles have discussed how isotretinoin interferes in skin healing without reaching any conclusion but suggesting that the overall risk is minimal or absent [10]. It is known that systemic isotretinoin use can cause skin thinning and inhibition of sebaceous glands [11]. Polarized views are explained by these two factors: 1) Due to its effect on sebaceous glands, isotretinoin was associated with the risk of poor healing of partial-thickness wounds, and 2) skin thinning caused by isotretinoin could increase the risk of inadvertent skin damage deeper than usual during dermabrasion [12]. On another hand, it was reported that cartilage grafts neighbouring the skin may become visible or extrude after the patient starts to use systemic isotretinoin following surgery which was the case of our patient [13]. Furthermore, the TORP procedure involves a physically thin epidermis of the external auditory canal, which makes it more susceptible to the effects of isotretinoin. No previous data describing the effect on the healing of donor or receptor sites of cartilage following the use of isotretinoin. Regarding our topic of interest, Allen BC et al. did a retrospective chart review that suggested the association between the use of isotretinoin post-rhinoplasty and the formation of nasal tip deformities, where 3 patients diagnosed with nasal tip asymmetries following rhinoplasty (before 2–2.5 years) and the postoperative use of isotretinoin which was similar to our case [13]. Patients stated adverse modifications in the appearance of their nose within 6 months after isotretinoin was started. The authors suggested 2 hypotheses: isotretinoin use caused the thinning of the nasal tip skin by its interactions with skin collagen or fibroblasts in the healing dermis resulting in increased contracture, or by accelerating the "shrinkwrap" phenomenon of postoperative healing. Several reviews have assessed the safety and pre and postoperative complications associated with systematic isotretinoin (Table 1). Recommendations on the use of isotretinoin before and after surgical procedures vary considerably, and most of them are based on insufficient clinical data. Although it is evident that isotretinoin has an impact on various healing processes, the magnitude of that impact appears to be overestimated. The body of evidence challenges the current clinical practice of delaying elective surgeries 6–12 months after isotretinoin exposure. However, literature still lacks evidence on isotretinoin effect on surgeries involving skin or cartilage grafts and whether the graft thickness, location, types, mode of surgery can be affected or not. Further research in these areas are needed which will positively improve patient outcome and safety.

**Table 1:** Relevant literature summary of recommendations on the use of Isotretinoin before and after surgical procedures.

Author	Title	Design	Summary
Allen BC et al. (2005) [13].	Complications Associated with Isotretinoin Use After Rhinoplasty	Retrospective analysis of case series.	Isotretinoin use after rhinoplasty was linked to nasal tip deformities including bossa formation, asymmetry, and prominence of a composite graft in three cases.
Miziołek B et al. (2019) [14].	The safety of isotretinoin treatment in patients with bone fractures	Review	Isotretinoin treatment may slow down the healing process in long bone fractures or cause bone thinning and osteoporosis. Accordingly, they recommend stopping isotretinoin or at least reducing its dose to the minimum.
Yahyavi S et al. (2020) [15].	Analysis of the Effects of Isotretinoin on Rhinoplasty Patients	Prospective Quasi-experimental design	Isotretinoin had no noticeable effect on the healing of rhinoplastic incisions and interior nose tissues., none had hypertrophy tissues or cartilaginous abnormalities, and the recovery was good.
Spring L K et al. (2017) [16].	Isotretinoin and Timing of Procedural Interventions: A Systematic Review With Consensus Recommendations	Review	Insufficient evidence to support delaying manual dermabrasion, superficial chemical peels, cutaneous surgery, laser hair removal, and fractional ablative and non-ablative laser operations, while mechanical dermabrasion and fully ablative laser procedures are not recommended with Isotretinoin systematic therapy.
McDonald KA et al. (2017) [17].	A Systematic Review on Oral Isotretinoin Therapy and Clinically Observable Wound Healing in Acne Patients	Systematic review	Inadequate data to support or oppose delaying elective procedures in people taking isotretinoin for acne. Clinicians must evaluate the risk of an adverse event vs the severity of the patient's acne scarring for each individual patient.
Tolkachjov NS et al. (2017) [18]	Surgical outcomes of patients on isotretinoin in the perioperative period: A single-center, retrospective analysis	Retrospective analysis of medical records	No differences in wound healing or abnormal scarring across the groups. Findings contradict the current practice of discontinuing isotretinoin use 6–12 months before surgery.
Chandrashekar BS et al.(2014) [19].	Safety of performing invasive acne scar treatment and laser hair removal in patients on oral isotretinoin: a retrospective study of 110 patients	A comparative, retrospective study	No Atypical scarring, delayed wound healing, keloids, or hypertrophic scars in patients who had the procedure. For patients taking oral isotretinoin, invasive procedures can be considered without compromising the outcome.
Ungarelli LF et al. (2015) [9].	Is It Safe to Operate on Patients Taking Isotretinoin?	Review	Data are limited to drawing conclusions on the effects of preoperative isotretinoin use on skin healing. Also, they suggested that surgeries involving skeletal muscles flaps are highly exposed to risks of muscle damage and necrosis.
Mahadevappa OH (2016) [20].	Surgical Outcome in Patients Taking Concomitant or Recent Intake of Oral Isotretinoin: A Multicentric Study-ISO-AIMS Study.	Multicentric cross-sectional study.	operating dermatosurogical and laser interventions in those who are receiving or have recently received isotretinoin is safe and that the present guidelines for avoiding such interventions should be studied further.

## 4 Conclusion

In this report, we describe a rare case of what seems an interference of isotretinoin with cartilage healing in which isotretinoin use may have been a factor in the development of prosthesis extrusion through cartilage graft after cartilage tympanoplasty and TORP. With the increased use of isotretinoin these days, otologists should be aware of the possibility of complications associated with isotretinoin use either intraoperative as thinning of tempanomeatal flap and tympanic membrane and possible postoperative complication as extrusion.

## Sources of funding for your research

This research received no external funding

## Ethical approval

Not applicable as this paper does not contain any studies on human or animal subjects.

## Consent

Written informed consent was obtained from the patient for publication of this case report and accompanying images. A copy of the written consent is available for review by the Editor-in-Chief of this journal on request.

## Author contribution

All authors have contributed substantially to study concept, design, literature review and draft manuscript preparation. All authors reviewed and approved the final version of this manuscript.

## Registration of research studies

1. Name of the registry: *Not applicable*
2. Unique Identifying number or registration ID: *Not applicable*
3. Hyperlink to your specific registration (must be publicly accessible and will be checked): *Not applicable*

## Guarantor

Mohammed Alwabili.

Ma.alwabili@gmail.com

malwabili@psmmc.edu.sa00966500812730.

Department of otolaryngology head and neck surgery.

Prince sultan military medical city.

Address:

Makkah Al Mukarramah Branch Rd, As Sulimaniyah, Riyadh 12233 11159, Saudi Arabia.

## Research registration

N/a.

## Provenance and peer review

Not commissioned, externally peer-reviewed.

## Declaration of competing interest

All authors declare no conflict of interest.
